# ONT-LibQC: rapid quality control of chromatin assay libraries using shallow nanopore sequencing

**DOI:** 10.64898/2026.10.19.760194

**Published:** 2026-10-24

**Authors:** Hongjiang Liu, Jongmin J. Kim

## Abstract

A next-generation sequencing (NGS) library for genomic assays can have an acceptable DNA concentration and size distribution yet lack the biological signal of interest. For chromatin assays, such as CUT&RUN and Micro-C, such problems often become apparent only after deep Illumina sequencing, delaying troubleshooting and protocol optimization. Here, we present ONT-LibQC, a workflow that uses a few thousand Oxford Nanopore Technologies (ONT) sequencing reads to examine finished libraries before deep sequencing. It reconstructs library molecules, identifies adapters and indexes, and assesses fragment sizes and the enrichment of biological signal. For CUT&RUN libraries, shallow, approximately 3,000 ONT reads were sufficient to show enrichment of promoter-associated H3K4me3, gene-body H3K36me3 and broad H3K27me3 profiles, consistent with profiles from Illumina deep sequencing. For Micro-C libraries, cis-contact fractions from shallow ONT sequencing closely matched Illumina results. Furthermore, full-length ONT reads revealed internal ligation structure and enabled estimation of the lengths of MNase digested DNA fragments. ONT profiles were reproducible across four technical replicate sequencing runs of each of two H3K4me3 libraries. Downsampling analysis further showed that key quality control (QC) features remained informative at lower read counts as low as 1,000 reads. Together, these results show that shallow ONT sequencing can provide rapid feedback on library quality, helping researchers identify potential problems before committing to deeper sequencing. ONT-LibQC is available through a web portal, a local graphical interface and a command-line workflow.

